# Account of a Case in Which a Cherry-Stone Was Extracted from an Abscess in the Abdomen

**Published:** 1781-11

**Authors:** 


II. *Account of a caſe in which a cherry-ſtone was extracted from an abſceſs in the abdomen. Communicated by* F. Swediar, M. D. *Phyſician in London. Read* October 22, 1781.

THE writings of practical authors afford a variety of inſtances of extraneous ſubſtances being taken into the ſtomach, and afterwards diſcharged from the ſurface of the body by abſceſs. All theſe caſes are proofs of the wonderful reſources of nature, and of courſe are worthy of being recorded. The following addition to the hiſtories of this ſort will perhaps not be deemed unworthy the notice of the Society.

 The

The patient was a young woman twenty-two years of age, and feemingly of a good habit of body. She enjoyed an uninterrupted ftate of good health till about the middle of Auguft 1779, when fhe was attacked with an excruciating colic, which continued for fome hours, but at length yielded to purgative medicines, glyfters, and opiates. It was not long, however, before fhe had a fecond attack of the fame kind, but with increafed violence. Her abdomen was painful when touched, particularly about the navel; fhe had frequent inclinations to vomit, and her pulfe indicated confiderable inflammation. A blifter was applied to the abdomen, and this with bleeding, the warm bath, and glyfters foon removed the vomiting and abated the pain; after which fhe took a folution of manna and Rochelle falt, and foon afterwards an opiate. Thefe medicines had the defired effect of procuring ftools and removing the pain.

This painful and alarming diforder continued to return from time to time, with greater or lefs violence, till the latter end of November, when the patient perceived a fmall painful fwelling near the navel, the fkin around which was evidently inflamed. At the end of another week this this tumour ſeemed to have an evident tendency to ſuppuration, which was encouraged by the application of a bread and milk poultice. Care was taken at the ſame time to obviate any feveriſh heat by keeping the patient's body open, and by a ſtrict attention to regimen. In about three weeks the abſceſs burſt ſpontaneouſly, and diſcharged about an ounce of pus. The next day a ſlight appearance of fœces was obſerved on the dreſſings, and the wound continued painful and inflamed. The uſe of the poultice was continued over the dreſſings; and as the pulſe was much quickened, in conſequence, as was ſuppoſed, of the inflammation, ſix ounces of blood were taken from the arm.

The patient complaining of a pricking ſenſation within the wound; a probe was introduced, and a hard ſubſtance being felt near the orifice, the opening was dilated with a common biſtoury, and an extraneous body extracted, which proved to be a cherry-ſtone almoſt wholly incruſted with fœces, but with a ſharp part of it projecting ſufficiently to account for the pain and irritation the patient had experienced.

After this ſubſtance was taken out, more fœces paſſed through the wound than before; but in the courſe of a few days the inflammation fubfided, and the difcharge leffened, fo that in the fpace of about feven weeks the wound was perfectly healed, and the patient has fince remained well.

*Newman Street,*

